# The effects of chronic stretch training on musculoskeletal pain

**DOI:** 10.1007/s00421-025-05747-9

**Published:** 2025-03-09

**Authors:** Andreas Konrad, Masatoshi Nakamura, Mahta Sardroodian, Nazanin Aboozari, Saman Hadjizadeh Anvar, David G. Behm

**Affiliations:** 1 Institute of Human Movement Science, Sport and Health, Graz University, Mozartgasse 14, 8010 Graz, Austria; 2 School of Human Kinetics and Recreation, Memorial University of Newfoundland, St. John’s, Newfoundland and Labrador Canada; 3 Faculty of Rehabilitation Sciences, NishiKyushu University, 4490-9 Ozaki, Kanzaki, Saga 842-8585 Japan; 4 Recovery and Performance Laboratory, Faculty of Medicine, Memorial University of Newfoundland, St. John’s, Newfoundland and Labrador Canada

**Keywords:** Stretching, Flexibility, Range of motion, Stretch tolerance, Stiffness

## Abstract

**Purpose:**

One of the primary mechanisms for the increase in range of motion following stretching is an increase in pain/stretch tolerance. However, it remains unclear whether stretching can reduce pain in patients suffering from musculoskeletal pain. Therefore, the purpose of this systematic review was to investigate whether chronic stretch training can decrease pain in patients suffering from musculoskeletal pain.

**Methods:**

In our search, we included three databases (PubMed, Scopus, and Web of Science) and after removing duplicates, screened 797 papers. Six papers were found to be eligible for this review. The inclusion criteria were controlled or randomized controlled trials that involved any type of chronic stretch training with participants experiencing musculoskeletal pain and where at least one pain output parameter was reported (e.g. visual analogue scale).

**Results:**

Of the six studies reviewed, four focused on the effects of stretching interventions on pain in patients, while the other two examined pain prevalence during the stretching period. The interventions lasted between 4 weeks and 6 months and involved either static or dynamic stretching techniques with in total 658 participants. Five of the six studies reported a significant decrease in pain scores or a reduction in the prevalence or severity of pain following the observation period.

**Conclusion:**

The findings indicate that stretching can alleviate pain by enhancing range of motion and reducing muscle stiffness, which may ease nerve pressure and lower muscle spindle activity. Although results were somewhat mixed, the evidence overall supports stretching as an effective intervention for relieving musculoskeletal pain.

## Introduction

Musculoskeletal pain affects a significant portion of the population. According to the Global Burden of Disease Study, musculoskeletal disorders have seen a 30% increase from 1990 to 2019 (Vos et al. 2020; Behm et al. 2021b). The World Health Organization (WHO) provides further insights into musculoskeletal conditions, noting that approximately 1.71 billion people are affected worldwide. Among these disorders, low back pain stands out with a prevalence of 568 million people, making it the main contributor to disability globally, with low back pain being the primary cause of disability in 160 countries (Cieza et al. 2020; Behm et al. 2021b). Hence, it should be a goal to counteract musculoskeletal pain, since it decreases the quality of life of the individual (Taylor 2005) and also causes an immense global economic burden (Blyth et al. 2019). One approach to overcoming musculoskeletal pain is muscle-strengthening activities such as resistance training (Owen et al. 2020; Hayden et al. 2021). Others have also suggested various forms of flexibility training (e.g., stretching, foam rolling, instrument assisted soft tissue mobilization, yoga) (Aboodarda et al. 2015; Behm 2018; Behm et al. 2021b) or movement re-education could be beneficial in reducing either acute or chronic musculoskeletal pain and discomfort (Taylor 2005; Cieza et al. 2020).

Considering flexibility training, muscle stretch training has been reported as an effective strategy. Stretching, whether through various techniques, has been found to acutely increase the range of motion (ROM) of a joint following a single stretching session (Behm et al. 2023), as well as chronically after several weeks of stretch training (Konrad et al. 2023a). Notably, a single static stretching exercise not only affects the ROM of the targeted joint but also influences other non-adjacent joints, leading to enhanced global flexibility (Behm et al. 2021a). These changes in non-stretched body regions were mainly attributed to an acutely enhanced global stretch tolerance (i.e., pain perception or increased pain pressure threshold) (Behm et al. 2021a). However, considering chronic stretch training, with the exception of contralateral effects (Nakamura et al. 2022), only local changes in pain tolerance were reported as one mechanism for the increase in ROM (Behm et al. 2021b; Nakamura et al. 2021; Konrad et al. 2023b). Other mechanisms underlying stretch-induced chronic pain reduction may be related to reduction in stress and stiffness of myofascial meridians, and reflex-induced increases in parasympathetic nervous activity (Behm et al. 2021b). It is important to note that these studies primarily involved participants who were not experiencing any chronic pain syndromes.

Therefore, a critical area of investigation pertains to whether stretch training can alleviate musculoskeletal pain. Given that a prior narrative review from Behm et al. (2021b) reported a potential decrease in musculoskeletal pain, but did not involve a systematic search, it is essential to emphasize the underdeveloped nature of the literature on the effect of stretching on pain. This underscores the significance of conducting a systematic review to provide a more quantitative assessment to comprehensively review the impact of stretch training on musculoskeletal pain and to address the current gaps in existing knowledge.

## Methods

This review was conducted according to the PRISMA guidelines and the suggestions from Moher et al. (2009) for systematic reviews with meta-analysis. The review was registered in PROSPERO with the code CRD42024536663.

### Search strategy

To identify all the relevant studies, we searched for eligible papers published until May 3rd 2024. Similar to the previous studies (Konrad et al. 2022, 2023a) the electronic literature search was performed in PubMed, Scopus, and Web of Science with the use of AND and OR Boolean operators. To find the eligible stretching studies the search term stretch* was used. Moreover, to assess studies on pain the following search terms were included: “muscle pain”) OR (“skeletal pain”) OR ("musculoskeletal pain") OR ("back pain") OR ("Knee pain") OR ("hip pain") OR ("ankle pain") OR ("shoulder pain") OR ("elbow pain") OR ("wrist pain"))) AND (chronic OR training OR long-term)) OR ABS (stretch*) AND ("muscle pain") OR ("skeletal pain") OR ("musculoskeletal pain") OR ("back pain") OR ("Knee pain") OR ("hip pain") OR ("ankle pain") OR ("shoulder pain") OR ("elbow pain") OR ("wrist pain"). To assess chronic studies, we additionally used the following keywords: chronic OR training OR long-term. The systematic search was conducted by two independent researchers. Initially the articles were screened by their title and then abstract. If the content remained unclear, the full text was retrieved for further screening and identifying the relevant papers. Following this independent screening process, the researchers compared their findings. The disagreements were resolved by jointly reassessing the studies against the eligibility criteria.

### Inclusion and exclusion criteria

This review considered studies that investigated the training effects of stretching on pain variables in participants’ musculoskeletal pain with or without chronic disease on pain parameters such as scales (visual analogue scale (VAS)), indexes (Oswestry Disability index), and questionnaires (Cornell Musculoskeletal Disorders Questionnaire, Standardized Nordic Questionnaire, Roland Morris questionnaire, Northwick Park Neck Pain Questionnaire and Short Form-36. The studies were included when they were either randomized controlled trials or controlled trials with an intervention duration ≥ 2 weeks (Freitas et al. 2018). This implied that we excluded studies which were dealing with the acute effects of stretching (or interventions shorter than < 2 weeks), investigated any combined treatment (e.g., stretching combined with strength training), or had another treatment as control condition. Moreover, we excluded review papers, case reports, special communications, letters to the editor, invited commentaries, conference papers, or theses.

### Extraction of the data and analysis

From the included papers, the characteristics of the participants (i.e., age, sex), sample size, characteristics of the intervention (i.e., total intervention duration in seconds, weeks of intervention) and results of the main variables (pain parameters) were extracted. For the main variables pre- and post-intervention values plus standard deviations (if available in the manuscript) were extracted. If some of the required data were missing in the included studies, the authors of the studies were contacted via email or similar channels (e.g., Research Gate). Where possible, effect sizes (i.e., Cohen’s d) were calculated for pre- to post-comparisons with G*Power (Faul et al. 2009) software assuming a conservative correlation between pre- and post-tests of 0.5 (Wilke et al. 2020). Cohen’s d was interpreted with values under 0.2 as indicating no or very small effect (trivial), 0.2 to less than 0.5 as a small effect, 0.5 to less than 0.8 as a moderate effect, and values of 0.8 or greater as a large effect (Cohen 1988).

Although a meta-analysis would have provided more quantifiable data, meta-analyses are primarily conducted on continuous data, whereas the identified studies in this review primarily utilized scales and questionnaires. While it may be possible to conduct a meta-analysis with ordinal and scale outcomes, the scales, indexes, and questionnaires used in these studies were diverse, making comparisons or analysis problematic. Hence, a systematic review only was incorporated to this research question.

### Risk of bias assessment and methodological quality

The methodological quality of the included studies was assessed using the PEDro scale. In total, 11 methodological criteria were rated by two independent researchers (AK and MN) and were assigned with either one or no point. Hence, higher scores indicated better methodological quality of the study. In the case of conflict between the two researchers, the methodological criteria were reassessed and discussed.

## Results

### Results of the search

**Fig. 1:**
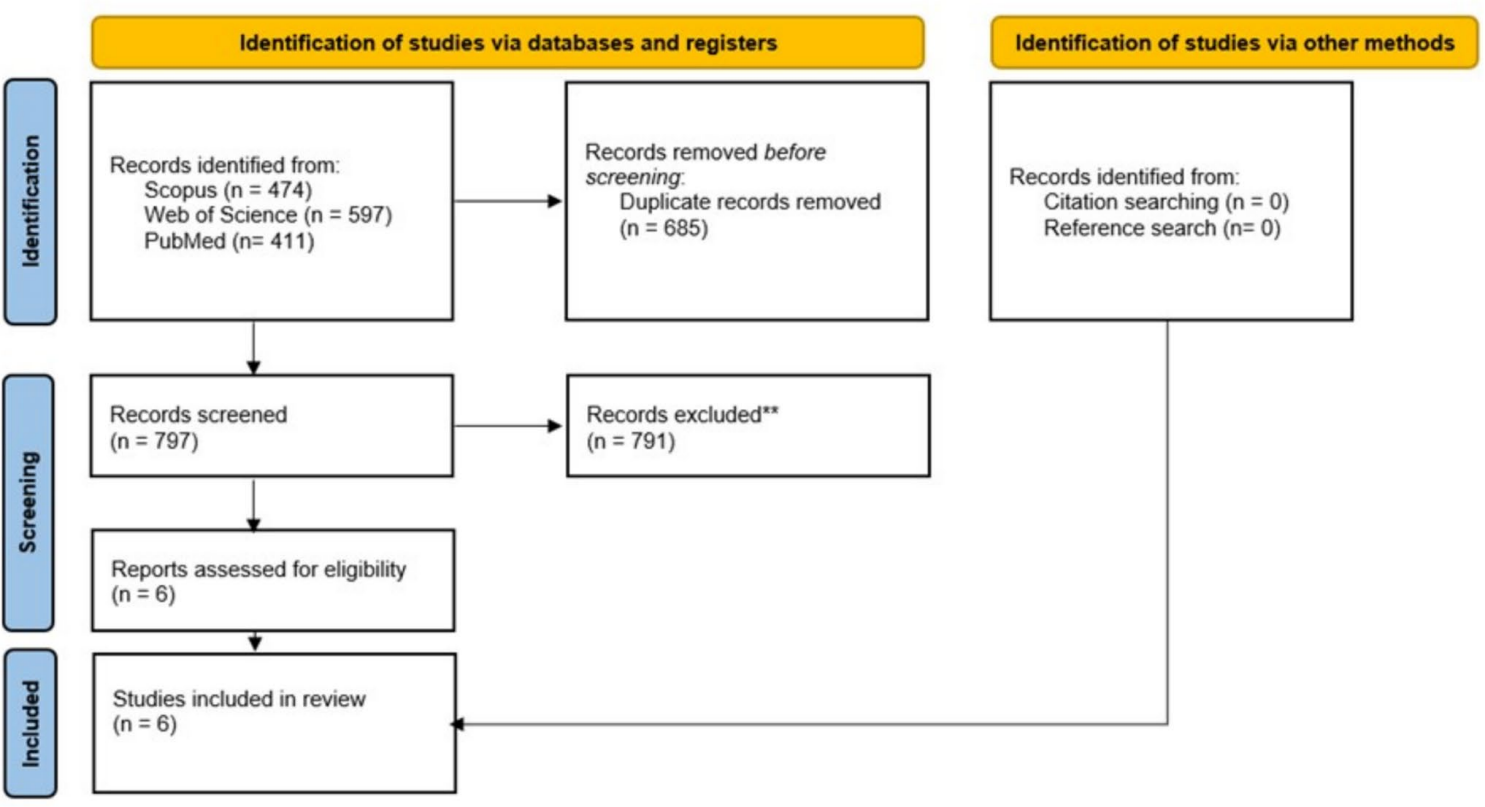
Prisma flow chart

Overall, after removal of the duplicates, 797 papers were screened, from which six papers were found to be eligible for this review. During the additional search of the references (search through the reference list) and citations (search through Google Scholar) of the six already included papers, no further papers were identified as relevant. Figure 1 shows the search process.

The methodological quality, as assessed with the PEDro scale (Table 1), revealed a range of scores between 7 and 10 points (out of 10) for all the included studies. The average PEDro score value was 7.5 (± 1.2), indicating a low risk of bias (Maher et al. 2003).

**Table 1:** PEDro scale criteria

Study	1^a^	2	3	4	5	6	7	8	9	10	11	Total
Ghasemi et al. (2020)	1	1	1	1	0	0	0	1	1	1	1	7
Tunwattanapong et al. (2016)	1	1	1	1	0	0	0	1	1	1	1	7
Holzgreve et al. (2021)	1	1	1	1	0	0	0	1	1	1	1	7
Shariat et al. (2018)	1	1	1	1	0	0	0	1	1	1	1	7
Shamsi et al. (2022)	1	1	1	1	1	1	1	1	1	1	1	10
Nye et al. (2021)	0	1	1	1	0	0	0	1	1	1	1	7

(1) Eligibility criteria were specified. (2) Subjects were randomly allocated to groups (in a crossover study, subjects were randomly allocated an order in which treatments were received). (3) Allocation was concealed. (4) The groups were similar at baseline regarding the most important prognostic indicators. (5) There was blinding of all subjects. (6) There was blinding of all therapists/researchers who administered the therapy/protocol. (7) There was blinding of all assessors who measured at least one key outcome. (8) Measures of at least 1 key outcome were obtained from more than 85% of the subjects initially allocated to groups. (9) All subjects for whom outcome measures were available received the treatment or control condition as allocated or, where this was not the case, data for at least 1 key outcome were analyzed by “intention to treat.” (10) The results of between-group statistical comparisons were reported for at least 1 key outcome. (11) The study provided both point measures and measures of variability for at least 1 key outcome. 1 = yes; 0 = no. a = Was not counted for the final score

An overview of the six eligible studies of the percentage change including effect sizes of the pre- to post-test comparisons is shown in Table 2. Additionally, Table 3 shows the main results of the statistics within the individual studies.

**Table 2:** Characteristics of the eligible studies

Study	Participants and study design	Stretch training characteristics	Outcome measure and pre- to post-test results (percentage change and effect size)								
Ghasemi et al.(2020)	92 male truck drivers (age: 36.70 ± 9.16) with chronic LBP were divided into: 1.) Stretch group (*n* = 31), 2.) Rest group (*n* = 30), 3.) Control group (*n* = 31)	12 weeks stretching with NR technique, 2–3 times per working day for 5 × 30 s/muscle. Muscles stretched were abdominal and lower back muscles	Stretch group: VAS: − 51.6% (*d* = 2.0); OLBPDQ: − 27.3% (*d* = 3.98); RMQ: − 67.3% (d = 2.68) Rest group: VAS: − 25.9% (*d* = 1.64); OLBPDQ: − 23.9% (*d* = 1.44); RMQ: − 37.2% (d = 1.61) Control group: VAS: − 11.4% (*d* = 0.46); OLBPDQ: − 10.0%(*d* = 0.39); RMQ: − 20.1% (d = 0.67)								
Tunwattanapong et al. (2016)	96 male and female office workers with moderate-to-severe neck pain were divided into 1.) Stretch group (*n* = 48; age: 36.5) and 2.) Control group (*n* = 48; age: 34.2)	4 weeks dynamic stretching, 5 times a week for 2 times a day with 20–30 repetitions/session of neck, shoulder stretching, shoulder rolling, trunk stretching, and back extension exercises Both groups received a brochure for proper position and ergonomics to be applied during daily work	Stretch group: VAS: − 32.8% (*d* = 1.39); NPNPQ: − 25.0% (*d* = 0.61); SF-36 physical: 24.1% (*d* = 0.65); SF-36 mental: 13.6% (*d* = 0.43) Control group: VAS: − 9.7% (*d* = 0.38); NPNPQ: − 8.2% (*d* = 0.18); SF-36 physical: 8.1% (*d* = 0.27); SF-36 mental: 2.0% (*d* = 0.07)								
Holzgreve et al. (2021)	252 male and female office workers (age: 44 ± 22) were divided into 1.) Stretch group (*n* = 156) and 2.) Control group (*n* = 96)	12 weeks of static stretching for 22 to 24 sessions with 5 exercises (i.e., whole body stretches) with 2 × 20 s	Nordic questionnaire for pain prevalence Stretch group*: Neck: − 26%; Shoulder: − 20%; Elbows: − 2%; Wrist/hands: − 24%; Upper back: − 56%; Lower back: − 23%; Thighs: − 28%; Knees: − 21%; Feet/ankles: − 54% Control group*: Neck: − 20%; Shoulder: − 13%; Elbows: + 147%; Wrist/hands: + 20%; Upper back: − 1%; Lower back: − 7%; Thighs: − 29%; Knees: − 1%; Feet/ankles: − 2%								
Shariat et al. (2018)	142 male and female office workers aged 20–50 years old with neck, shoulders, and lower back pain were divided 1.) Stretch group (*n* = 43), 2.) Ergonomic group (*n* = 37) 3.) Combined (*n* = 34) 1.) and 2.), and 4.) Controls (*n* = 28)	Six months of dynamic stretching for 3/week, 3 × 13 exercises with 10 repetitions. Locations of the stretched tissue were NR	Cornell Musculoskeletal Disorders Questionnaire was used								
				Stretch group* (%)		Ergonomic group* (%)		Combined group* (%)		Control group* (%)	
			Neck	− 86		− 84		− 82		− 20	
			Right shoulder	− 91		− 84		− 87		− 28	
			Left shoulder	− 88		− 86		− 82		− 16	
			lower back	− 89		− 80		− 85		− 1	
Shamsi et al. (2022)	45 male and female chronic nonspecific LBP patients were divided into 1.) Static stretching group (*n* = 15; age: 37.7 ± 9.0) 2.) Strength training group (*n* = 15; age: 37.1 ± 13.4), 3.) Control group (*n* = 15; age: 39.1 ± 11.6)	Four weeks, 3/week 3 × 2 min static hamstring stretch Additionally, all groups received conventional physiotherapy including e.g., transcutaneous electrical stimulation and heat pack	Stretch group: VAS: − 53.9% (*d* = 1.73); ODI: − 62.3% (*d* = 1.71) Strength training group: VAS: − 58.5% (*d* = 2.86); ODI: − 53.7% (*d* = 1.81) Control group: VAS: − 57.5% (*d* = 2.0); ODI: − 45.3% (*d* = 1.48)								
Nye et al. (2021)	31 male and female senior dental hygiene students (age: 21 to 22) were divided into 1.) Stretch group (*n* = 15) and 2.) Control group (*n* = 16)	10.5 weeks of static stretching before every clinical session. Exercises were aiming neck, shoulders, back, and hands and wrists with no details reported except that the whole exercise lasted 5—7 min	Standardized Nordic Questionnaire for pain prevalence								
			Pain sums		5 weeks		*d*		10.5 weeks		*d*
			Stretch group		24%		0.22		14%		0.13
			Control group		126%		0.83		95%		0.65
			Overall pain		5 weeks		*d*		10.5 weeks		*d*
			Stretch group		− 9%		0.16		− 9%		0.16
			Control group		− 7%		0.17		− 10%		0.25
			Neck pain		5 weeks		*d*		10.5 weeks		*d*
			Stretch group		− 18%		0.25		− 12%		0.16
			Control group		0%		0		33%		0.53
			Shoulder pain		5 weeks		*d*		10.5 weeks		*d*
			Stretch group		− 8%		0.09		− 18%		0.18
			Control group		− 32%		0.29		− 21%		0.19
			Hands/wrist pain		5 weeks		*d*		10.5 weeks		*d*
			Stretch group		− 18%		0.18		− 18%		0.17
			Control group		− 9%		0.13		− 23%		0.3
			Low back pain		5 weeks		*d*		10.5 weeks		*d*
			Stretch group		− 16%		0.14		− 25%		0.21
			Control group		− 17%		0.19		− 39%		0.48

*LBP* lower back pain, *NR* not reported in the study, *VAS* visual analogue scale concerning pain, *OLBPDQ* Oswestry low back pain disability questionnaire, *ODI* Oswestry disability index, *RMQ* Roland Morris questionnaire, *NPNPQ* northwick park neck pain questionnaire, *SF*–36 short form-36, * no effect size calculation could have been performed according to the data in the manuscript

**Table 3:** Statistical results of the individual studies

Study	Main outcome
Pain patients	
Ghasemi et al. (2020)	Low back pain Stretching * > controls Rest * > controls Stretching * > rest
Tunwattanapong et al. (2016)	Neck pain Stretching * > controls ≥ 3 × stretching/week * > < 3 × stretching/week
Shariat et al. (2018)	Neck, shoulder, and lower back pain Stretching * > controls
Shamsi et al. (2022)	Low back pain Stretching = strength training Stretching = controls Strength training = controls
Pain prevalence	
Holzgreve et al. (2021)	Stretching * > controls
Nye et al. (2021)	5 weeks: stretching * > controls 10.5 weeks: stretching = controls

“* > ” = a statistically significant favor or better effect; **“ = ”** = same effect or no statistical significant difference

Out of the six studies, four investigated the effects of a stretch intervention to counteract pain in pain symptomatic patients (Tunwattanapong et al. 2016; Shariat et al. 2018; Ghasemi et al. 2020; Shamsi et al. 2022), while the remaining two studies explored pain prevalence throughout a stretch intervention period (Nye et al. 2021; Holzgreve et al. 2021). The stretch intervention duration varied between 4 weeks and 6 months and either the static or dynamic stretching technique was used. The scales used to explore pain in the total 658 participants of which 308 participants performed a stretch training were the VAS, Oswestry low back pain disability questionnaire, Roland Morris questionnaire, Northwick Park Neck Pain Questionnaire, Nordic questionnaire, physical as well as mental dimension of the Short Form-36, Cornell Musculoskeletal Disorders Questionnaire, and Oswestry Disability Index.

The various studies demonstrated significant small to large (Tunwattanapong et al. 2016), moderate to large (Nye et al. 2021), or large magnitude (Ghasemi et al. 2020; Shamsi et al. 2022) decreases in pain according to the survey questionnaires. However, there was also frequent pain attenuation in the control groups albeit with smaller magnitudes of improvement (Tunwattanapong et al. 2016; Ghasemi et al. 2020) or same magnitudes of improvements (Shamsi et al. 2022). In three out of those four cases, the decrease in pain or pain prevalence was significantly better in the stretching groups compared to the control group (Tunwattanapong et al. 2016; Ghasemi et al. 2020; Nye et al. 2021).

In two studies it was not possible to calculate effect sizes based on the data in the studies. However, both reported a significantly more pronounced decrease in pain in the stretch groups compared to the controls (Shariat et al. 2018; Holzgreve et al. 2021).

## Discussion

The major findings of this systematic review were that with the six studies that fit the inclusion criteria, 658 participants performed stretch training programs either statically or dynamically over a range of 4 weeks to 6 months reporting significant improvements (decreases) in pain or a decrease in pain prevalence in five out of the six studies. The changes in the levels of pain were determined with scales (VAS), indexes (Oswestry Disability index), and questionnaires (Cornell Musculoskeletal Disorders Questionnaire, Standardized Nordic Questionnaire, Roland Morris questionnaire, Northwick Park Neck Pain Questionnaire and Short Form-36).

Shamsi et al. (2022) was the only study that reported no favorable effect of stretch training compared to the control condition or a strength training group on alleviating lower back pain. However, it has to be mentioned that all three groups performed a traditional therapy such as transcutaneous electrical stimulation or heat pack but also strength and stretch training for the spinal and abdominal muscles. Consequently, all three groups had large magnitude improvements in both VAS and Oswestry Disability Index to a similar extent. Hence, it can be assumed that the additional stretch program of the hamstring muscles did not have an additive effect and might have not caused a further improvement in musculoskeletal pain. According to a meta-analysis by Hori et al. (2021), it was not possible to conclude that impaired flexibility would cause lower back pain. Consequently, the increased ROM of the hamstrings in the participants of Shamsi et al. (2022) which has likely occurred following both the additional stretch training (Konrad et al. 2023a) and strength training (Alizadeh et al. 2023) was likely not enough to induce changes in pain levels.

Interestingly, Nye et al. (2021) showed a significant large effect size decrease in pain in the stretching group compared to the control group in pain prevalence following 5 weeks of stretching but not following 10.5 weeks. However, although the favorable effect was not significant following the 10.5 weeks of stretching compared to the controls, the effect size indicated a medium magnitude of change. As only 15 participants were in the stretch group and 16 participants in the control group it can be assumed that the study was slightly underpowered which might explain the near significant difference (*P* = 0.07). The authors speculated that another reason might be that the participant’s compliance with the stretch protocol decreased slightly in the second half of the study and hence, the change was not significant anymore. Another explanation might be that a plateau effect has occurred following > 5 weeks of stretching. The participants of Nye et al. (2021) were asked to perform the same stretch exercises with the same durations throughout the 10.5 weeks. However, a progressive load as performed in previous studies (Panidi et al. 2021) might have caused a further improvement in flexibility and hence, possibly in musculoskeletal pain as well (Arendt-Nielsen et al. 2011). On the other hand, Tunwattanapong et al. (2016) reported that participants who stretched for ≥ 3/week induced significant better improvements in neck pain compared to participants who performed < 3/weeks. This would indicate a dose–response effect at least up to 4 weeks.

Lately, various studies have shown that high-volume static stretch training with weekly stretch durations of ~ 60 min per muscle group could induce improvements in muscle strength as well as increase muscle mass (Panidi et al. 2021; Warneke et al. 2022, 2023a,b; Wohlann et al. 2023). It is known that lower limb muscle strength is impaired in patients with low back pain (Santana De Sousa et al. 2019). Resistance training which can improve the strength and function of the surrounding connective tissue has been associated with decreases in pain perception (Knutzen et al. 2007; Baker et al. 2001; Hughes et al. 2004). High volume stretching might be an appropriate stimulus to decrease musculoskeletal pain by its reported increase in muscle strength.

**Fig. 2:**
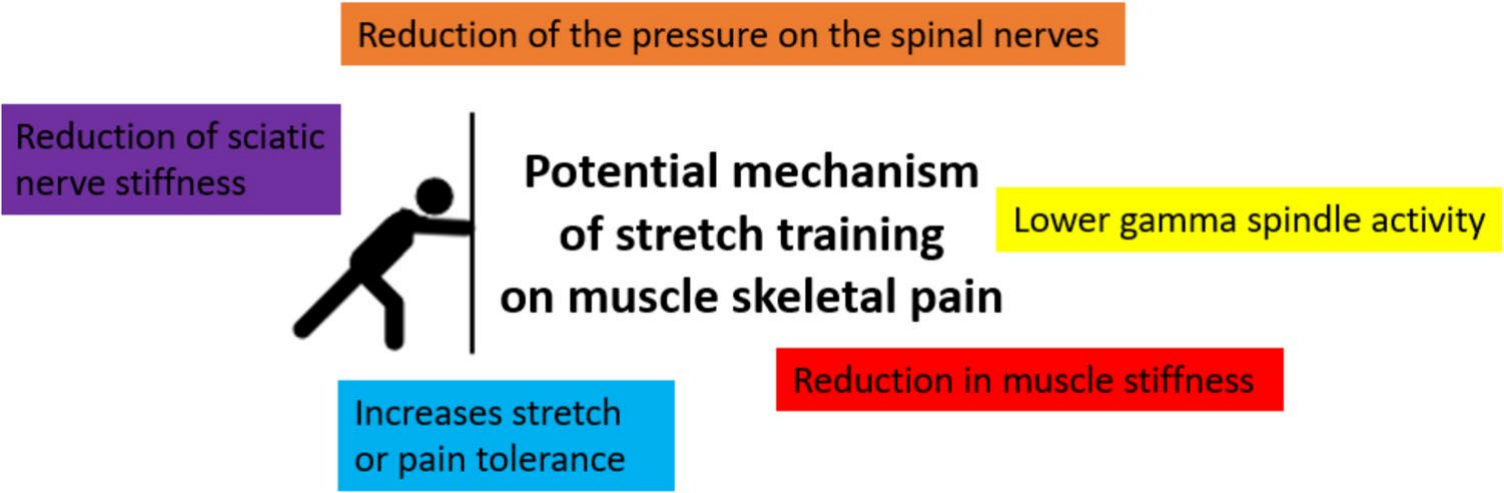
Potential mechanism of stretch training on muscle skeletal pain

Considering the potential mechanism of stretching on muscle skeletal pain (see also Fig. 2), Shariat et al (2018) suggested stretching can help relieve pressure on the spinal nerves. They assumed that this mainly occurs due to the increased ROM, particularly in the hip extensors, flexors, and the piriformis muscle. Increased flexibility can improve hip/pelvis orientation positively affecting spinal nerves, alleviating back pain and compensatory overuse syndromes (Behm 2018). Reduction in sciatic nerve stiffness following stretch training was reported by Andrade et al. (2020) which can decrease pain levels, especially in the lower limbs. Moreover, Shamsi et al. (2022) suggested that lower back pain increases the activity of sensory receptors in the back’s soft tissue, leading to greater gamma spindle activity and muscle stiffness to keep the spine stable. A recent meta-analysis reported that stretch training for several weeks can decrease muscle stiffness (Takeuchi et al. 2023) and hence, according to the assumption of Shamsi et al. (2022) would likely lead to an attenuation of pain in the lower back. Since stretching does not just increase the compliance of muscle tissue but also connective tissue as well, decreased stiffness of myofascial meridians may also contribute to decreasing stressful tension on nociceptors. In addition to muscle, fascia and skin are also highly innervated by sensory neurons (Schleip et al. 2003). Even Shamsi et al. (2022) performed paraspinal and abdominal muscle stretching and strengthening as a conventional treatment in all participants of the study (i.e., stretch group, strengthening group, control group) and showed an improvement in pain levels. Increased muscle and tendon compliance (decreased stiffness) could aid in the absorption of forces from external perturbations allowing potential painful stressors to the musculoskeletal system to be attenuated (Behm 2018; Behm et al. 2021b). On the other hand, the reported strength increases with prolonged static stretch training could enhance the stability of painful joints. Stretch-induced strength/stability adaptations may seem counterintuitive to the prior claims of stretch-induced increases in tissue compliance. However, both can work in concert as muscle strength and stability improvements would be an active anticipatory and ongoing response to stressful forces whereas decreased muscle and connective tissue stiffness (increased compliance) would be a more passive component helping to absorb external perturbations.

One major mechanism for the increase in range of motion of a joint following chronic stretching is adjusted stretch or pain tolerance of the stretched tissue (Konrad and Tilp 2014). Even, unilateral stretching can cause increased stretch and pain tolerance in the non-stretched contralateral homologous muscle/joint (Nakamura et al. 2022). Consequently, it can be assumed that local and contralateral homologous nociceptors of the stretched limb decrease pain sensitivity and might decrease pain chronically as well. According to this theory, it can be suggested that e.g., knee pain might improve if nociceptors are stimulated by frequently stretching the quadriceps femoris and hamstring muscles.

Future research should take high volume stretching into account that might also increase muscle strength and hence, likely further lead to an improvement in pain. Additionally, to the best knowledge no controlled study has performed stretch training in patients with musculoskeletal pain in the limbs (e.g., knee pain, shoulder pain). Hence, this should be taken into account for future research. Additionally, there is a need for studies that investigate the effect mechanisms to better understand the change in musculoskeletal discomfort/pain following stretch training.

## Conclusion

In conclusion, this systematic review found that stretching programs, whether static or dynamic, can lead to significant reductions in pain or pain prevalence in individuals over periods ranging from 4 weeks to 6 months. Five out of six studies reported improvements, as measured by various pain scales and questionnaires. The findings suggest that stretching may help relieve pain by increasing the ROM and reducing muscle stiffness, which could alleviate pressure on nerves and decrease muscle spindle activity. Despite some mixed results, evidence supports the use of stretching as a beneficial intervention for musculoskeletal pain. Future research should explore the effects of stretching on limb-specific musculoskeletal pain and investigate the underlying mechanisms to better understand how stretching influences pain relief.

## Data Availability

The original contributions presented in the study are included in the article. Further inquiries can be directed to the corresponding author.

## Abbreviations

ROM: Range of motion
VAS: Visual analogue scale
